# Phenotypic Characteristics of Hyperacusis in Tinnitus

**DOI:** 10.1371/journal.pone.0086944

**Published:** 2014-01-31

**Authors:** Martin Schecklmann, Michael Landgrebe, Berthold Langguth, the TRI Database Study Group

**Affiliations:** 1 Department of Psychiatry and Psychotherapy, University of Regensburg, Regensburg, Germany; 2 Department of Psychiatry and Psychotherapy, Social Foundation Bamberg, Bamberg, Germany; UNLV, United States of America

## Abstract

**Background:**

Many people with tinnitus also suffer from hyperacusis. Both clinical and basic scientific data indicate an overlap in pathophysiologic mechanisms. In order to further elucidate the interplay between tinnitus and hyperacusis we compared clinical and demographic characteristics of tinnitus patients with and without hyperacusis by analyzing a large sample from an international tinnitus patient database.

**Materials:**

The default dataset import [November 1^st^, 2012] from the Tinnitus Research Initiative [TRI] Database was used for analyses. Hyperacusis was defined by the question “Do sounds cause you pain or physical discomfort?” of the Tinnitus Sample Case History Questionnaire. Patients who answered this question with “yes” were contrasted with “no”-responders with respect to 41 variables.

**Results:**

935 [55%] out of 1713 patients were characterized as hyperacusis patients. Hyperacusis in tinnitus was associated with younger age, higher tinnitus-related, mental and general distress; and higher rates of pain disorders and vertigo. In relation to objective audiological assessment patients with hyperacusis rated their subjective hearing function worse than those without hyperacusis. Similarly the tinnitus pitch was rated higher by hyperacusis patients in relation to the audiometrically determined tinnitus pitch. Among patients with tinnitus and hyperacusis the tinnitus was more frequently modulated by external noise and somatic maneuvers, i.e., exposure to environmental sounds and head and neck movements change the tinnitus percept.

**Conclusions:**

Our findings suggest that the comorbidity of hyperacusis is a useful criterion for defining a sub-type of tinnitus which is characterized by greater need of treatment. The higher sensitivity to auditory, somatosensory and vestibular input confirms the notion of an overactivation of an unspecific hypervigilance network in tinnitus patients with hyperacusis.

## Introduction

Chronic tinnitus (ringing in the ears) is a widespread disorder affecting 5–15% of the population [1], [2]. 10% of tinnitus patients are severely distressed and experience a distinct decline in their quality of life [1], [2]. Concomitant symptoms are sleep disturbance, anxiety, depression, irritation, and concentration difficulties [3]. Furthermore, 40% of patients with tinnitus suffer from hyperacusis [4] while 86% of patients with hyperacusis have concomitant tinnitus [4]. Hyperacusis is described as hypersensitivity to sounds or the perception of ordinary sounds as louder as normal and with uncomfortable intensity [4]–[6].

The large overlap in prevalence between tinnitus and hyperacusis suggests that the two disorders share common pathophysiological mechanisms and risk factors. For tinnitus, hearing loss represents the main risk factor. It is assumed that the reduced peripheral auditory input causes alterations of the neural activity along the auditory pathway [7]. In addition, non-auditory areas of attention allocation, emotional processing and memory encoding are involved in the generation and maintenance of tinnitus [8], [9]. The most efficient tinnitus therapies are tinnitus retraining and cognitive behavioural therapy by which quality of life can be significantly improved [10]. There is only very limited information available about hyperacusis. However, existing literature shows further similarities for both conditions in addition to the overlap in prevalence. Hyperacusis is also associated with hearing loss [11] and there is evidence for altered neural activity in auditory [12] and non-auditory cortical areas [13], [14]. For both conditions, there is only a low correlation between audiometric measures and subjective ratings by visual analogue scales or questionnaires [15]. Due to these similarities, the same therapeutic approaches of tinnitus retraining and cognitive behavioural therapy have been suggested as treatment for hyperacusis [4], [16].

Despite a clear overlap between tinnitus and hyperacusis, prevalence rates also indicate a population of patients who suffer from only one of the two conditions. A simplified definition of tinnitus is a “constant existent sound without external source” while hyperacusis is a “consistently exaggerated or inappropriate response to sounds that are neither threatening nor uncomfortably loud to a typical person” [17]. Thus, tinnitus might be related to increased spontaneous activity along the auditory pathway and hyperacusis to increased stimulus-related activity [12]. It is an open question if both hyperactive states are based on the same neurobiological condition [18], [19], however there is clear clinical evidence that there is no complete overlap between tinnitus and hyperacusis. For example, hyperacusis, but not tinnitus, is a typical symptom of Williams syndrome, a hereditary disorder characterised by abnormal serotonergic metabolism [4]. There is also evidence that tinnitus and hyperacusis differ from each other in neural activity in the auditory pathway, specifically in the cingulate and orbitofrontal cortex [14], [20].

Based on these observations one could postulate that the existence of comorbid hyperacusis defines a distinct subtype of chronic tinnitus. In this case one would expect that tinnitus patients with comorbid hyperacusis differ in their clinical and demographic characteristics from tinnitus patients without hyperacusis. Thus, the aim of the present study was the systematic evaluation of tinnitus patients with and without hyperacusis with respect to demographic, audiometric, and tinnitus characteristics, as well as tinnitus-related and general distress. High ecological validity was pursued by investigating a large sample from an international database [21].

## Materials and Methods

The data presented in this study were derived from the Tinnitus Research Initiative (TRI) Database [22]. Data management was conducted according to the TRI Data Handling Plan (TRI-DHP V07, May 9^th^, 2011). Data analysis was performed according to the TRI Standard Operating Procedure (TRI-SA V01, May 9^th^, 2011), thereby following a study-specific Statistical Analysis Plan (SAP-009, Nov 22^th^ 2012) that was written according to the TRI SAP template (TRI-SAP V01, May 9^th^, 2011). All documents can be accessed at http://database.tinnitusresearch.org/. Analysis details can be found at the end of the methods section.

The default dataset import (November 1^st^, 2012) from the TRI Database consisted of patients who were treated between 2005 and 2012 at tinnitus centres worldwide. Patients gave written informed consent to record their data in the database and for inclusion in analysis. The project was approved by the local ethics committee (Ethikkommission der Fakultät für Medizin der Universität Regensburg) at the location of the database, the University of Regensburg, Germany.

Hyperacusis was defined by the question “Do sounds cause you pain or physical discomfort?” of the Tinnitus Sample Case History Questionnaire (TSCHQ) [22]. Patients who did not answer this question (n = 620) or answered it with “I don’t know” (n = 187) were excluded from analysis resulting in a sample of 1713 patients (“yes”: n = 935; “no”: n = 778). Thus, 55% of the patients were categorized as suffering from comorbid hyperacusis, which is a higher rate than indicated in the literature (40 (40%) [4]). Assessment was performed before the first consultation in the tinnitus clinics and included demographic and tinnitus-related questions (TSCHQ), questionnaires with respect to tinnitus, depression, and quality of life, and tinnitus-related numeric rating scales. An overview of all included variables can be seen in table 1. We also included audiology data (mean hearing level, minimal masking level, and tinnitus pitch), which were assessed at the first consultation in the different participating tinnitus centers. Mean hearing level was indicated by dB HL (hearing level) over all frequencies (0.125, 0.25, 0.5, 1, 2, 3, 4, 6, and 8kHz) in both ears; tinnitus loudness by minimal masking level (minimal volume of white noise to predominate the tinnitus percept); and the frequency of the tinnitus was assigned using pitch matching (for details see [23]). If no data were available from the screening visit (first consultation) we used data from the baseline visit of a clinical intervention. If both screening and baseline data were available we used the mean of the data at both time points.

**Table 1:** Descriptive values and statistics of group contrasts.

	tinnitus	tinnitus+hyperacusis	statistics
sample characteristics			
**current age (years)**	**54.3±12.9 (n = 759)**	**50.7±13.2 (n = 927)**	**T = 5.640; df = 1648; p<0.001; d = 0.275**
**age at tinnitus onset (years)**	**45.3±13.9 (n = 736)**	**41.6±13.7 (n = 883)**	**T = 5.352; df = 1617; p<0.001; d = 0.266**
gender (female/male)	245/533 (n = 778); 32/68%	366/569 (n = 935); 39/61%	χ^2^ = 10.840; df = 1; p<0.001; d = 0.161
tinnitus characteristics			
duration (months)	98.6±108.3 (n = 720)	100.6±106.2 (n = 876)	T = −0.364; df = 1594; p = 0.716; d = 0.018
tinnitus laterality (unilateral-right/unilateral-left/elsewhere)	114/148/513 (n = 775); 15/19/66%	96/161/675 (n = 932); 10/17/72%	χ^2^ = 9.824; df = 2; p = 0.007; d = 0.152
pulsatile (no/yes)	650/118 (n = 768); 85/15%	714/204 (n = 918); 78/22%	χ^2^ = 12.727; df = 1; p<0.001; d = 0.175
day-to-day changes of loudness (no/yes)	339/430 (n = 769); 44/56%	354/570 (n = 924); 38/62%	χ^2^ = 5.782; df = 1; p = 0.016; d = 0.116
manifestation over time (intermittent/constant)	121/650 (n = 771); 16/84%	109/819 (n = 928); 12/88%	χ^2^ = 5.609; df = 1; p = 0.018; d = 0.114
onset (gradual/abrupt)	381/373 (n = 754); 50/49%	425/458 (n = 883); 48/52%	χ^2^ = 0.937; df = 1; p = 0.333; d = 0.048
character (tone/noise/crickets/other)	415/153/132/68 (n = 768); 54/20/17/9%	522/160/161/81 (n = 924); 57/17/17/9%	χ^2^ = 2.014; df = 3; p = 0.570; d = 0.024
**onset related event (none/one/multiple)**	**149/515/113 (n = 777); 19/66/15%**	**96/596/241 (n = 933); 10/64/26%**	**χ^2^ = 49.836; df = 2; p<0.001; d = 0.347**
modulating factors			
maskable by music or sound (no/yes)	173/489 (n = 662); 26/74%	192/628 (n = 820); 23/77%	χ^2^ = 1.458; df = 1; p = 0.227; d = 0.062
**somatic modulation (no/yes)**	**566/206 (n = 772); 73/27%**	**562/355 (n = 917); 61/38%**	**χ^2^ = 27.341; df = 1; p<0.001; d = 0.256**
**influence by noise (no/yes)**	**388/257 (n = 645); 60/40%**	**142/654 (n = 796); 18/82%**	**χ^2^ = 274.378; df = 1; p<0.001; d = 0.969**
influence by nap (worsens/reduces/no effect)	115/41/573 (n = 729); 16/6/79%	144/99/622 (n = 865); 17/11/72%	χ^2^ = 17.811; df = 2; p<0.001; d = 0.100
influence by sleep (no/yes/don’t know)	354/129/277 (n = 760); 47/17/36%	324/220/357 (n = 901); 36/24/40%	χ^2^ = 23.349; df = 2; p<0.001; d = 0.155
**influence by stress (worsens/reduces/no effect)**	**423/11/316 (n = 750); 56/2/42%**	**718/4/188 (n = 910); 79/0/21%**	**χ^2^ = 97.530; df = 2; p<0.001; d = 0.490**
hearing and tinnitus matching			
**hearing level (dB HL mean of all frequencies)**	**25.6±13.9 (n = 596)**	**21.3±14.7 (n = 698)**	**T = 5.374; df = 1292; p<0.001; d = 0.299**
wearing of hearing aids (no/yes)	675/91 (n = 766); 88/12%	788/130 (n = 918); 86/14%	χ^2^ = 1.906; df = 1; p = 0.167; d = 0.068
subjective hearing problems (no/yes)	327/440 (n = 767); 43/57%	308/613 (n = 921); 33/67%	χ^2^ = 15.067; df = 1; p<0.001; d = 0.189
minimal masking level (dB HL)	54.6±20.1 (n = 429)	56.6±23.2 (n = 505)	T = −1.394; df = 932; p = 0.164; d = 0.091
tinnitus loudness	61.6±23.6 (n = 745)	66.7±27.7 (n = 894)	T = −3.959; df = 1637; p<0.001; d = 0.196
tinnitus pitch (Hz)	6329±3107 (n = 436)	6669±3491 (n = 514)	T = 1.574; df = 948; p = 0.116; d = 0.102
**subjective tinnitus pitch (low, medium, high, very high)**	**33/177/382/171 (n = 763); 4/23/50/22%**	**15/155/478/272 (n = 920); 2/17/52/30%**	**χ^2^ = 27.545; df = 3; p<0.001; d = 0.250**
concomitant complaints and therapies			
**suffering from headache (no/yes)**	**526/242 (n = 768); 68/31%**	**492/414 (n = 906); 54/46%**	**χ^2^ = 35.095; df = 1; p<0.001; d = 0.293**
**suffering from temporomandibular joint** **complaints (no/yes)**	**640/126 (n = 766); 84/16%**	**672/236 (n = 908); 74/26%**	**χ^2^ = 22.321; df = 1; p<0.001; d = 0.232**
**suffering from neck pain (no/yes)**	**400/365 (n = 765); 52/48%**	**351/565 (n = 916); 38/62%**	**χ^2^ = 32.909; df = 1; p<0.001; d = 0.283**
**suffering from any other pain (no/yes)**	**526/236 (n = 762); 69/31%**	**486/421 (n = 907); 54/46%**	**χ^2^ = 41.389; df = 1; p<0.001; d = 0.318**
**suffering from vertigo (no/yes)**	**562/192 (n = 754); 74/25%**	**534/368 (n = 902); 59/41%**	**χ^2^ = 43.147; df = 1; p<0.001; d = 0.326**
**preceding tinnitus treatments (none/one/several)**	**155/133/490 (n = 778); 20/17/63%**	**121/138/676 (n = 935); 13/15/72%**	**χ^2^ = 19.728; df = 2; p<0.001; d = 0.215**
**current psychiatric treatment (no/yes)**	**696/80 (n = 776); 90/10%**	**738/186 (n = 924); 80/20%**	**χ^2^ = 30.820; df = 1; p<0.001; d = 0.273**
questionnaires			
**tinnitus questionnaire (0–84)**	**35.8±17.1 (n = 544)**	**44.3±17.8 (n = 816)**	**T = **−**8.683; df = 1358; p<0.001; d = 0.471**
**tinnitus handicap inventory (0–100)**	**41.9±22.3 (n = 755)**	**53.6±23.0 (n = 918)**	**T = **−**10.466; df = 1671; p<0.001; d = 0.512**
**Beck depression inventory (0–63)**	**9.3±8.0 (n = 716)**	**12.8±9.4 (n = 867)**	**T = **−**7.922; df = 1581; p<0.001; d = 0.398**
**quality of life: physical health (4–20)** *	**15.0±3.0 (n = 572)**	**13.9±3.1 (n = 631)**	**T = 5.913; df = 1201; p<0.001; d = 0.341**
**quality of life: psychological functions (4–20)** *	**14.4±2.7 (n = 570)**	**13.5±2.9 (n = 634)**	**T = 5.648; df = 1202; p<0.001; d = 0.326**
quality of life: social relationships (4–20)*	14.7±3.0 (n = 568)	14.3±3.3 (n = 633)	T = 2.143; df = 1199; p = 0.032; d = 0.123
quality of life: environmental factors (4–20)*	15.8±2.6 (n = 572)	15.6±2.4 (n = 633)	T = 1.474; df = 1203; p = 0.141; d = 0.085
rating scales			
loudness (0–10)	6.1±2.3 (n = 752)	6.6±2.2 (n = 903)	T = −3.933; df = 1653; p<0.001; d = 0.193
**discomfort (0–10)**	**6.7±2.4 (n = 753)**	**7.3±2.2 (n = 906)**	**T = **−**5.110; df = 1657; p<0.001; d = 0.251**
**annoyance (0–10)**	**6.3±2.5 (n = 753)**	**6.9±2.4 (n = 908)**	**T = **−**5.285; df = 1659; p<0.001; d = 0.260**
**ignorability (0–10)**	**6.4±2.8 (n = 754)**	**7.0±2.6 (n = 907)**	**T = **−**4.770; df = 1659; p<0.001; d = 0.234**
**unpleasantness (0–10)**	**6.3±2.5 (n = 753)**	**6.9±2.4 (n = 99)**	**T = **−**5.058; df = 1660; p<0.001; d = 0.248**

Meaningful contrasts are defined by p<0.001 and d>0.2 and marked in bold font.

* High scores mean high functioning in quality of life in contrast to other questionnaires and rating scales.

For statistical analyses we contrasted tinnitus patients with and without hyperacusis using two-sided Student t-tests for continuous variables (e.g., age) and chi-square-tests for categorical variables (e.g., gender). In order to correct for the high number of dependent variables, significance threshold was set to a level of 0.001. We also reported the effect size Cohen’s d [24]. Due to reasons of uniformity, chi-square Pearson r was transformed into Cohen’s d by using the Statistical calculation spreadsheet “Converting effect sizes” at http://www.stat-help.com/based on a published converting formula [25]. Effect sizes are indicated as small with a range from 0.2 to 0.5, medium with values between 0.5 and 0.8, and high with values above 0.8. We relate the presentation of the results to significant effects (p<0.001) with at least small effect sizes since only these effects are considered as clinically meaningful.

## Results

A detailed overview of the raw data and statistics is given in table 1. The result section is segmented according to the subgroups of variables as specified in table 1. There were significant differences between tinnitus patients with and without hyperacusis for age at tinnitus onset and age of treatment (small effect sizes). Patients with hyperacusis were younger (4 years on average). There was a significant effect for sex, but with a negligible effect size.

For many tinnitus-related characteristics we did not find significant group contrasts: tinnitus duration, laterality, character, day-to-day changes in loudness, intermittent/constant manifestation over time, or gradual/abrupt onset. However, patients with hyperacusis had a significantly higher probability to have pulsatile-like tinnitus, but with a negligible effect size. Spontaneous occurrence of tinnitus (without triggering events at tinnitus onset) was significantly less frequent in patients with hyperacusis (with small effect size).

For tinnitus-modulating factors, there were significant differences for all investigated variables except the ability to mask the tinnitus using music or sounds. The influence of sleep time and nap time was significant, but with negligible effect sizes. Hyperacusis patients could more frequently modulate their tinnitus with somatic maneuvers such as head movements, more frequently had a stress-sensitive tinnitus (all small effect sizes), and their tinnitus was more often influenced by noise (high effect size).

With respect to hearing function, hyperacusis patients had better hearing level in the pure tone audiometry (small effect size), but the patients’ subjective assessment of hearing function was worse (significant, negligible effect size). A similar discrepancy between psychoacoustic measurements and subjective reports was observed for tinnitus pitch and loudness. Whereas minimal masking levels and tinnitus pitch match did not differ between groups, tinnitus patients with hyperacusis reported higher loudness ratings (significant, negligible effect size) and higher subjective pitch (significant, small effect size) of their tinnitus. Also in numeric rating scales, hyperacusis was related to higher tinnitus loudness, but also to reduced ability to ignore tinnitus and to increased discomfort, annoyance and unpleasantness (all with small effect sizes except for loudness which had negligible effect size).

With respect to comorbidities, hyperacusis was more frequently associated with headache, temporomandibular joint (TMJ) complaints, neck pain, general pain syndromes, and vertigo (all with small effect sizes). Hyperacusis was also related to an increased number of tinnitus treatment attempts and a current psychiatric treatment (both with small effect sizes).

Increased scores were found in the tinnitus questionnaire, the tinnitus handicap inventory, and the depression scale (small or medium effect sizes) for hyperacusis patients. In line, quality of life was significantly reduced for these patients with small effect sizes in the physical and psychological domain whereas there were no significant differences for quality of life in social relationships or environmental factors.

## Discussion

**Figure 1:**
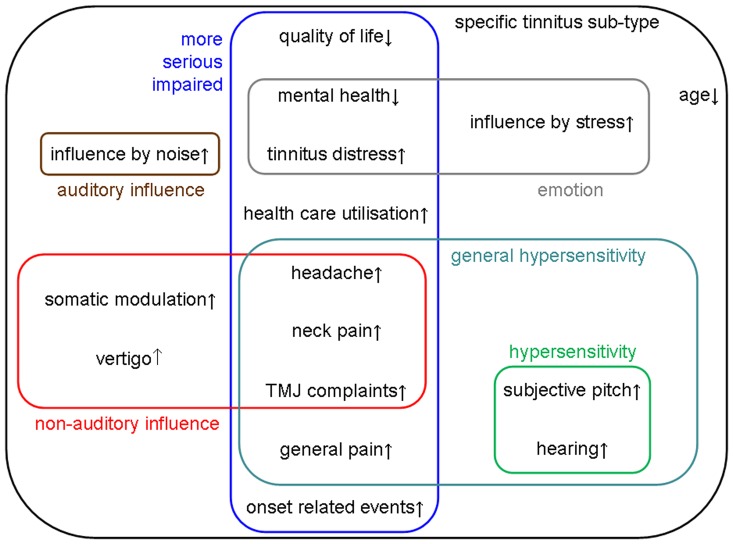
Illustrative overview of the Tinnitus Research Initiative Database analysis showing significant [p<0.001; d>0.2] effects for hyperacusis in tinnitus. Significant variables are subsumed for illustrative purposes in colored boxes.

We defined hyperacusis with the question ““Do sounds cause you pain or physical discomfort?”. The main finding of our database analysis is, that tinnitus patients with and without hyperacusis differ in a large number of characteristics. In figure 1, all significant findings with at least small effect sizes are shown and grouped into different domains. The biggest effect size was found for the variable ‘influence by noise’ (brown box, figure 1), which means that patients with hyperacusis (82%) showed a higher probability that their tinnitus is influenced by external sounds and noise in contrast to patients without hyperacusis (40%). The finding that in people with both hyperacusis and tinnitus the sensitivity to sounds is directly related to the perception of tinnitus is an indication for an overlap in the pathophysiological mechanisms, as previously proposed [18], [19], [26], [27]. Our finding also supports the notion of an abnormal increase of gain within the auditory system as a relevant mechanism for both tinnitus and hyperacusis [12], [26]. The sensitivity to sound in some but not all tinnitus patients may represent a clinical criterion for pathophysiologically distinct subtypes of this disorder and may have important implications for the interpretation of imaging studies of tinnitus, which use auditory stimulation paradigms [20], [28].

The next highest effect sizes were found for the questionnaires. Patients with comorbid hyperacusis showed increased scores in tinnitus and depression questionnaires and decreased scores in physical and psychological dimensions of quality of life. Along with this the numeric rating scales showed enhanced scores for tinnitus loudness, discomfort, annoyance and unpleasantness. The ability to ignore tinnitus was subjectively decreased in hyperacusis patients. These findings clearly confirm that comorbid hyperacusis increases the negative influence of tinnitus on quality of life [29], [30]. Moreover the risk for comorbid depression is increased as demonstrated by both an increased amount of depressive symptoms and more frequent psychiatric treatment. Hyperacusis patients screened for mental conditions showed a high probability for psychiatric comorbidity, especially anxiety disorders and neuroticism personality traits [31]. The higher overall number of tinnitus-related treatments, the higher number of reported events triggering tinnitus onset, and the high number of comorbid conditions can be summarized by the notion that tinnitus with hyperacusis is a more serious condition than tinnitus alone (blue box, figure 1).

Increased mental burden, increased tinnitus distress, reduced quality of life and increased modulation of tinnitus by stress in tinnitus patients with hyperacusis point to an increased role of brain circuits for emotional processing (grey box, figure 1) in patients with both hyperacusis and tinnitus. For tinnitus, there is clear evidence from neuroimaging studies of the involvement of emotion-related brain areas [8], [9], [32] in the pathology. For hyperacusis there are some suggestions of involvement of non-auditory areas. In a recent electroencephalography study, hyperacusis in tinnitus patients was related to increased activity in the auditory, anterior cingulate, and orbitofrontal cortex and increased connectivity between these areas [14]. The anterior cingulate and orbitofrontal cortex are brain areas which are related to vigilance and salience detection; pathalogically they are involved in anxiety disorders and may represent a “hypervigilance” or “hyperresponsive” network [14].

In addition to the increased relevance of emotional factors we also found evidence to suggest a more frequent involvement of somatosensoric aspects in tinnitus with comorbid hyperacusis (red box, figure 1). Increased prevalence of headache, neck pain, and TMJ complaints together with increased modulation of tinnitus by somatic manoeuvres all highlight the role of somatosensoric afferents. For TMJ disorder, there is clear evidence that TMJ complaints and tinnitus are inter-related. Severity of tinnitus is related to severity of TMJ pain [33]; tinnitus patients with subjectively or objectively assessed TMJ disorders can modulate their tinnitus with somatic manoeuvres [34], [35] and the prevalence of tinnitus is increased in TMJ disorder [36]. This trigeminal influence might be mediated via the cochlear nucleus [37]. Both the hypersensitivity to somatic input and the increased prevalence of vertigo in tinnitus patients with hyperacusis could be the consequence of a generally increased sensitivity to sensory input, independent from the sensory modality (turquois box, figure 1). This would be in line with the notion of an overactivation of an unspecific hypervigilance network in people with hyperacusis [14].

Besides hypersensitivity to auditory, somatic and vestibular input, a divergence between subjective estimation and audiologic measures of tinnitus pitch, loudness and hearing level was found (green box, figure 1). Hyperacusis patients with tinnitus rate their hearing level as worse, their tinnitus as louder, and their tinnitus pitch as higher whereas the audiologic measurements do not show such a tendency for tinnitus patients with hyperacusis. These findings fit to the generalized hypervigilance hypothesis [38] which states that these patients have a “perceptual habit that involves subjective amplification of a variety of aversive sensations” [39]. This hypothesis was deduced from studies in fibromyalgia patients who showed increased perceived intensity and unpleasantness of cutaneous and auditory stimuli [39]. The notion of an unspecific hyperresponsive network is further supported by the investigation of people with subjective electromagnetic hypersensitivity, those who more frequently perceive tinnitus [40] and those who demonstrate an overactivation of the hyperresponsive network even in the absence of a real sensory stimulus [41].

It should be noted that tinnitus characteristics like duration, laterality, character, day-to-day changes in loudness, or intermittent/constant manifestation showed no relationship with hyperacusis. A possible hyperacusis subtype of tinnitus is not related to or a consequence of specific tinnitus characteristics. This makes it probable that hyperacusis is not a consequence but rather a predisposition for tinnitus. This notion is supported by animal studies, which have demonstrated behavioural changes suggestive of the development of hyperacusis, before the presence of tinnitus, after noise trauma [42]. Hypersensitivity might be relevant both for external sounds and for intermittent and chronic tinnitus percepts that cause difficulties in coping with the tinnitus.

A limitation of our study is the definition of hyperacusis. In the lack of internationally accepted diagnostic standards we defined hyperacusis by the answer to the question “Do sounds cause you pain and physical discomfort?”. However, this question may focus particularly on one specific aspect of hyperacusis. Hyperacusis or decreased tolerance to sound might also exist without pain or physical discomfort. In addition, another aspect related to hyperacusis is the phenomenon of loudness recruitment which is the abnormally rapid increase in perceived loudness with increasing steps of presented sounds. Loudness recruitment is typically caused by sensorineural hearing loss and is restricted to frequencies of hearing loss [5], [6]. It is suggested that hyperacusis and loudness recruitment represent different conditions which are not necessarily exclusive [4]. Another aspect is the missing precision of sounds. The question might be ambiguously interpreted and related not only to external sounds but also to the tinnitus. Based on these limitations we suggest that future studies should clearly describe the used definition of hyperacusis and should clearly consider related aspects of hyperacusis such as loudness recruitment. Furthermore it has to be mentioned that the conclusions of this analysis are limited to patients with hyperacusis and tinnitus. We are well aware that hyperacusis may also be present without tinnitus, but we cannot draw any conclusions about hyperacusis without tinnitus from our manuscript.

All these findings together with the fact that hyperacusis is related to an earlier onset and younger age at presentation at the clinic with an average of four years, affirm the assumption that hyperacusis constitutes a specific tinnitus subtype (black box, figure 1). This subtype is characterized by higher tinnitus related and general distress, decreased mental and somatic health, increased influence of sensory inputs and general hypersensitivity. However, we cannot exclude that the observed differences between tinnitus patients with and without hyperacusis all reflect increased severity in the hyperacusis group rather than a distinct mechanism of generating tinnitus. Increased severity might mediate the effects found for the other variables. Future studies should try to control for such spurious correlations. Independent of the interpretation, these findings highlight the importance of identifying comorbid hyperacusis both for clinical management and for neuroscientific tinnitus research.
